# Urology during COVID-19 Pandemic Crisis: A Systematic Review

**DOI:** 10.1055/s-0040-1722341

**Published:** 2021-01-15

**Authors:** Bikash Bikram Thapa, Dhan Shrestha, Sanjeeb Bista, Suresh Thapa, Vikram Niranjan

**Affiliations:** 1 Department of Surgery, College of Medicine, Nepalese Army Institute of Health Sciences, Kathmandu, Nepal; 2 Department of Emergency Medicine, Mangalbare Hospital, Morang, Nepal; 3 Health Research Institute/Graduate Entry Medical School, University of Limerick, Limerick, Ireland

**Keywords:** COVID-19, infection, pandemic, urology, standard urology care

## Abstract

**Background** Coronavirus disease 2019 (COVID-19) has evolved as a pandemic of unimaginable magnitude. The health care system is facing a tremendous challenge to provide ethical and quality care. The transformation of the patient-based care to population-based care during the COVID-19 pandemic has raised ethical dilemma among urologists. Our objective is to explore the consensus in modified standard urology care, that can be adopted and applied during COVID-19 and similar pandemic.

**Methods** We adopted an exploratory study design using secondary data. The data were extracted from a web-based medical library using keywords “COVID-19,” “severe acute respiratory syndrome coronavirus 2 (SARS-CoV-2),” and “urology.” We identify and extrapolate (screening, eligibility, and inclusion) the data using PRISMA protocol, and summarize pandemic standard urology care under four main themes: (1) general urology care, (2) choice of surgical modality, (3) triage, and (4) urology training.

**Result** We identified 63 academic papers related to our research question. The majority are expert opinions and perspectives on urology care. The common consensus is triage-based urology care and surgeries. Life or organ threatening conditions need immediate attention. Universal protective measures (personal protective equipment, safe operative environment) and protocol-based patient care are necessary to prevent and control SARS-CoV-2 infection. Conservation of the resources and its rational distribution provide an ethical basis for population-based health care during a pandemic. Informed decision making serves best to patients, families, and society during the public health crisis.

**Conclusion** COVID-19 pandemic tends to transform standard urology practice into crisis standard population-based care. The consensus in crisis is drawn from evolving pieces of medical evidence and public health ethics. The provision of urology care during a pandemic is based on the availability of resources; severity of the disease, consequences of deferment of service, and dynamics of the pandemic.

The occurrence and the outcomes of the COVID-19 (coronavirus disease-2019) pandemic have been unprecedented. Worldwide it has infected 4,864,881 people and has caused 321,818 deaths till May 20, 2020. 1 The health care system is confronting an ethical dilemma of scarce resource allocation, triage of care, and professional duty to care. 2 3 Majority of the non-COVID health problems have been neglected. Catering COVID-19, as well as non-COVID care, is part of robust hospital preparedness and response plan.

The relationship between COVID-19 and urology is multifaceted and dynamic. 4 The rapid surge in the COVID-19 cases has caused redistribution and reorganization of the health services (including urology). As the pandemic keeps evolving the impact becomes unpredictable and the benefit often becomes incomprehensible. 5 6 In the meantime, the essential and nondeferrable urology care needs a modified but rational approach. The basis of prioritization and duration of deferral, the consequences of deferral and delay, the success of alternative modes of medical and surgical treatment are largely unanswered. 6 The emerging and evolving scientific evidence helps formulating guidelines for urology case management. This systematic review aims to organize crisis standard evidence that would assist in informed decision making for a urologist, during COVID-and post-COVID pandemic era.

## Methods

**Fig. 1:**
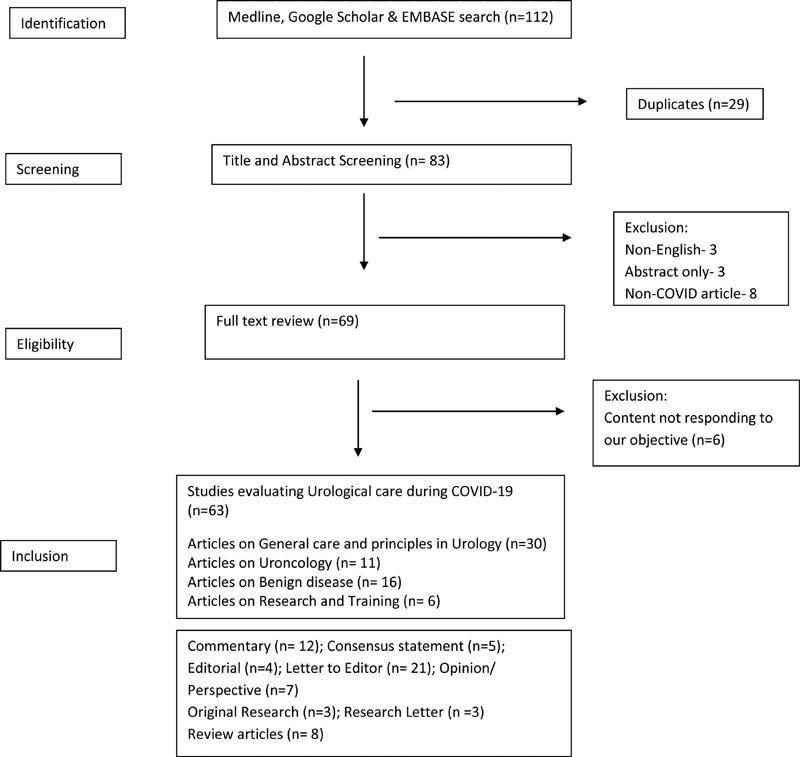
PRISMA Protocol for Systematic Review.

The present systematic review was conducted based on the PRISMA protocol ( Fig. 1 ). Online database till May 20, 2020 (Medline, Google Scholar, and EMBASE) was searched with keywords—“COVID-19,” “SARS-Cov-2,” coronavirus in combination with “Urology.” The titles and abstracts were independently reviewed by three authors to identify the potentially related articles. The full texts were reviewed and data summary (the type of article, place of publication, target topics heading in urology, and recommended guideline) were tabulated and result generated.

The studies included were about: (a) general urology care, (b) choice of surgical modality, (c) triaging urology care, and (d) training. The literature with inaccessible full-text and non-English research articles was excluded. “Pandemic standard urology care” and its guiding principles were tabulated.

## Results

Out of 63 academic articles, 30 papers are related to general urology care, 16 are about the benign urological condition, 11 about uro-oncology, and six about urology training. The majority of the articles are expert opinions and perspectives. Only 16 articles are original research papers. We grouped the research papers according to the objectives and the information were analyzed to summarize the recommendations. The current recommendation for pandemic standard urology care is drawn from limited scientific evidence. The new standard of care exclusively aims to prevent and control COVID-19 infection, protect occupational health, and optimize scarce resources by continuing the life and organ saving treatment and interventions. The consensus guidelines for crisis standard urology care based on the above principles keep evolving as this pandemic progress.

### General Urology Care

The health care system needs to set a clear plan of action in response to COVID-19 pandemic settings ( Table 1 ). Minimal risk exposure for the safety of health care workers, fair distribution and conservation of resources, strategic repurposing of the surgical services including operating theaters, and professional solidarity among health care workers are necessary to charter health care during a pandemic. 7 8 Reconfiguring elective surgeries conserves resources and provides extra space, stuff, and, staff for COVID care. 9 10 Urological care based on clinical condition and calculated risk provides an ethical basis of a treatment plan. Inter-department collaboration, transparent communication, and surge capacity contingency can address unforeseeable challenges during a pandemic. 9 Education and training prepares and motivates health care workers in duty. 5 9 Surgical staff can be kept reserve when not required. Telemedicine (patient consultation, multidisciplinary conference, and electronic database) is a viable option to mitigate the risk of exposure and contact-free care. 9

**Table 1:** General guidelines for urological care during COVID pandemic 4 5 7 8 9 11 14 15 16 20 23 27 29 52 53

• Ensure adequate essential personal protection equipment.
• Postpone elective/ nonessential surgeries.
• Reduce outpatient visit.
• Triage patients analyzing risk benefit and through informed decision making.
• Simplify diagnostic and staging process.
• Education and training of health care staff.
• Conservation of resources.
• Reorganization of operation room structure and function.
• Collaboration among surgical allied unit to find common solution.
• Prepare contingency plan or surge capacity to meet the emerging crisis.
• All operative cases should be tested for COVID.
• Separate COVID-19 positive patients from noninfected patients in each level of care.
• Procedures in local anesthesia or spinal block whenever possible.
• Equitable and fair distribution of operation room and the critical care resources.
• Adopt nonsurgical or open surgical procedure to laparoscopic procedure.
• Maintain negative room pressure and air filters during minimal invasive procedure.
• Surgeries should be performed by experienced surgeon outside learning curve.
• Avoid transrectal diagnostic procedure (ultrasound, digital rectal examination).
• Consider minimal hospital visit to patients.
• Defer all types of systemic chemotherapy.

Different levels of personal protective equipments are available for health care workers. Maximum utilization of the personal protective equipment can be done depending upon the various level of transmission risk (low, moderate, and high). 11 12 Operating on COVID-19 positive cases needs adequate planning, precaution, and execution. The importance of PPE (personal protective equipment), negative pressure room, minimal personnel movement, disinfection and sterilization, and viral filters was underscored in mitigating the risk. 4 13 14 15 Emergencies and oncology cases must be continued with judicious use of an operating room and strained critical care resources. 5 9 10 16 Reorganizing operation theater and rescheduling the surgeries depending upon the volume of cases, available resources, and expertise allow for efficient patient management and exposure risk reduction. 7 8

Infection prevention and control is the arduous task in health care settings. The key steps of the infection control during surgical care are: thorough hand washing, environmental cleaning, patient decolonization, vascular care, and surveillance. For environmental control, combination of deep cleaning with surface disinfectants using quaternary ammonium compound and alcohol is recommended. 17 Sufficient air exchanges (20 cycles per hour) are necessary to reduce the particles and viral load. 14 For patient decolonization, preoperative chlorhexidine wipes, two doses of nasal povidone-iodine within 1 hour of incision, and a chlorhexidine mouth rinse are recommended. 17 The standard of sterilization and disinfection for reprocessing instruments should not be compromised. All endoscopic instruments are subjected to “high-level disinfection” to kill severe acute respiratory syndrome corona virus-2 (SARS-CoV-2) that causes COVID-19 disease. 18

### Choice of Surgical Modality

The least invasive intervention modalities that can be completed in local or regional anesthesia will serve the best interest of patients and urologists. 10 14 Endoscopic surgery is considered relatively safe. However, the closed-system suction evacuation of irrigation fluid is required. Necessary precaution is taken not to cause iatrogenic trauma that could compromise endourological procedure and its outcome. Disposable equipment and supplies are preferred over reusable ones. 14 Aerosol generating procedure needs extra precaution and level III personal protection. Ultrasonic scalpels produce a large amount of water vapor, aerosols, and smoke formation. 19 Monopolar with inbuilt smoke evacuator provides protection. It is highly recommended to ensure measures to avoid body fluid spillage during surgery and measures to protect from it. 11 14 20 21 Frequent suction of smoke and use of ultralow particulate air filter (ULPA) are recommended. Pneumoperitoneum should be set at minimal acceptable pressure. The leak in pneumoperitoneum should be avoided and prevented. Balloon trocar can prevent air leak. Laparoscopic trocar and specimen need careful removal when pneumoperitoneum is still present. 21 The experts are skeptical about the use of two-way insufflations system like air seal that defy ULPA filter function. 14 Open surgeries are preferred over minimal invasive surgeries (laparoscopic and robotic) provided the risk does not outweigh the benefit. 19 22

### Triaging Urological Care

Triaging the urology services during a pandemic crisis has drawn the attention of more scholars. The principles guiding the urology care during the COVID-19 pandemic are listed in Table 2 . The prioritization and categorization of the urological care are enumerated in Table 3 . Experts have proposed prioritization of urological surgeries into five tiers 23 (0, emergency and 4, nonessential) or four tiers 11 [low, high, emergency, and nonessential]) to provide treatment triage during an ongoing pandemic. The decision of deferring or undertaking urological cases and surgeries should be guided by public health-focused ethical consideration. 16 23 Backed by informed decision, local context, and impact of the global pandemic on the health system one or the entire four tiers can be stopped, paused, or continued.

**Table 2:** Principles and ethical values behind urology care triage during pandemic 4 11 14 15 16 23 29 45 52

• Status of SARS-CoV-2 test and COVID-19 disease condition.
• Trend of pandemic in local context and calculated risk of exposure.
• Disease severity or stage.
• Prognosis by deferring.
• Available alternative treatment modalities.
• Resources (human, capital, and infrastructure) availability and utilization.
• Expected total hospital stay, recovery, and number of follow-up visit.
• Expected complications and strain to the critical care unit.
• Risk of exposure during referral or deferral.

Abbreviations: COVID-19, coronavirus disease 2019; SARS-CoV-2, severe acute respiratory syndrome coronavirus 2.

**Table 3:** Urology care triage and recommendation 5 11 16 22 23 29 31 32 38 39 40 42 43 44 45 49 50

Nondeferrable/Emergency/Life threatening	Deferrable for 3–6 mo	Active surveillance	Consider medical therapy (Chemo/Hormonal/Immunotherapy)	Deferred till pandemic ends or ≥6 mo
*Uro-oncology*				
1. Associated gross hematuria. 2. Ca penis (T2-T4). 3. Groin dissection in node positive carcinoma penis (<4 cm, mobile). 4. High-risk UTUC. 5. MIBC (T2-T3, Any *N* ). 6. High-risk bladder cancer. 7. RCC with hematuria and renal or IVC involvement. 8. Post chemotherapy RPLND. 9. Intravesical BCG in high-risk NMIBC.	1. Ca penis (Tis, Ta, T1). 2. Low-risk CaP. 2. Intermediate risk CaP. 3. High-risk CaP (consider neoadjuvant hormonal therapy). 4. Low-risk UTUC. 5. Low and intermediate risk bladder cancer. 6. Asymptomatic RCC. 7. Good and intermediate IMDC risk RCC (neoadjuvant target therapy). 8. Suspected adrenal tumor. 9. Intravesical therapy for low and intermediate NMIBC.	1. Groin negative ca penis (low and intermediate risk). 2. Fixed, >4 cm Groin node in carcinoma penis. 3. Testicular tumor (CS-I). 4. Selected low-risk CaP.	1.Fixed, >4 cm node in Ca penis-chemotherapy. 2. Testicular tumor (CS-IS, CSII, CS-III)-chemotherapy. 3.Metastatic CaP and CRPC—complete androgen blocked. 4.CRPC 5. Metastatic bladder cancer (consider immunotherapy). 6. Metastatic RCC-target therapy. 7. Wilm's tumor.	
*Urological emergencies* Testicular torsion, Fournier gangrene, obstructed uropathy (with or without infection), acute urinary retention, clot retention, urethral injury, penile fracture, trauma, infected prosthesis, and priapism.	*Outpatient procedures* Prostate biopsy, Office cystoscopy, pressure flow studies, stent, or nephrostomy change, intravesical therapy in low-risk bladder cancer.		Benign prostatic enlargement, Lower ureteral stone <10 mm.	**Non essential and low priority Elective Surgeries** Uncomplicated urolithiasis, Reconstructive surgeries, Surgery for infertility, prosthesis surgery, Surgeries of BEP
*Cadaveric renal transplant*	*Systemic chemotherapy*		**Living Donor Renal Transplant**
	*Benign disease* Ureteral stone without obstruction. PUJO with stable function. Recto/pubo urethral fistula		**Hands on Residency training**
*Pediatric urology:*				
Urosepsis with obstruction trauma with hemodynamically unstable, malignant testicular, or paratesticular tumor, rhabdomyosarcoma of bladder or prostate, testicular torsion, paraphimosis, obstructed hernia.	cryptorchidism, high-risk VUR. Progressive loss of function in PUJO and obstructed megaureter, PUV, stone disease with febrile UTI, Wilm's tumor.		High-risk Wilm's tumor-chemotherapy.	Hydrocele, inguinal hernia, circumcision, incontinence surgery, meatotomy, botulinum injections: hypospadias, buried penis, bladder augmentation and diversion. Pyeloplasty without loss of differential function, VUR, uncomplicated urolithiasis

Abbreviations: BCG, Bacillus Calmette–Guérin; BEP, benign enlargement of prostate; Ca, carcinoma; CaP, carcinoma prostate; CRPC, castrations resistant prostate cancer; CS, clinical stage; IMDC, International metastatic RCC database consortium; IVC, inferior vena cava; MIBC, muscle invasive bladder cancer; NMIBC, nonmuscle invasive bladder cancer; PUJO, pelviureteric junction obstruction; PUV, posterior urethral valve; RCC, renal cell carcinoma; RPLND, Retroperitoneal lymph node dissection; UTI, urinary tract infection; UTUC, upper tract urothelial carcinoma.

#### Outpatient Urology Care

The outpatient visits should be reduced to a minimum. Progressively deteriorating disease conditions or organ or life-threatening conditions are only recommended to visit the urology clinic. “Telehealth-integrated patient management protocol” is encouraged. 24 25 Virtual clinics have been proposed for the continuation of essential outpatient care. Radiological investigation and cystoscopy for an emergency condition like gross hematuria and urosepsis should not be deferred. 26

The office-based diagnostic procedures (flexible cystoscopy, pressure flow studies) for the benign condition can be deferred. The outpatient urological procedures like prostate biopsy follow-up cystoscopy, replacement of ureteral stents and nephrostomy tube, and intravesical therapy for low-risk nonmuscle invasive bladder cancer (NMIBC), and NMIBC patients who have already completed 1 year of maintenance therapy can be deferred. 16 23 27 28 However, the risk and benefit need to be evaluated with informed decision making. 5 11

#### Emergency Urological Care

Urological emergencies are either organ threatening or life threatening and cannot be deferred. Necessary personal protective measures are taken before embarking on interventions. Consideration is given to the least invasive procedure with a minimum operative time that can be conducted in the local or regional anesthetic block. The majority of these emergency urological conditions are minor surgeries, endourological, and percutaneous procedures done under local or regional anesthesia without delay. 11 23 29 Tefik et al advise to manage obstructing urolithiasis depending upon the status of renal function, infection, and pain. 30

#### Uro-Oncology

The majority of the urological malignancies are given high priority and the required intervention is strongly recommended. 16 23 29 31 Urological surgeries can be triaged on basis of the COVID-19 test, cancer stage, the outcomes of delaying, and the provision of alternative treatment modalities. 16 31 One-third to two-third of the planned oncology surgeries have been rescheduled during the COVID-19 pandemic. 11 22 23 31 Systemic chemotherapy is better avoided due to high risk of immunosuppression. Alternative therapies like immunotherapy, target therapy, and hormonal therapy are considered safer. Radiotherapy that requires repeated health care center visit is discouraged. 22 27 Intravesical therapy for bladder cancer is strictly limited to the high-risk groups. 11 28 32 Interventional diagnostic and follow-up procedures for the low-risk in terms of outcome (prostate biopsy, surveillance cystoscopy, and intravesical therapy) can be deferred safely. 5 11 29 Low-risk disease that otherwise can be taken for surgeries can be put on active surveillance. 22

#### Benign Urological Condition

Except for emerging complications, the majority of the benign urological condition can be postponed until the pandemic ends. 15 Nonurgent urolithiasis intervention can be deferred. But urolithiasis with symptoms and complications required urinary diversion or symptomatic relief. 30 All types of surgeries for benign prostatic enlargement (endoscopic and minimal invasive) are classified nonessential and the acute condition can be managed with temporary bladder diversion. 16 A serious concern is made by academician regarding men's health. 33 34 35 Witherspoon and colleagues recommended individual consideration for care of: male infertility, testicular mass, testicular pain, sperm banking, and testicular deficiency syndrome. 36 37

Elective renal transplant surgery is not advisable during the acute phase of the current pandemic. 15 38 Ritschl et al 39 has elaborately mentioned about the recommendation for both donor and recipients of solid organ transplant including kidney. Complete epidemiological, clinical, and laboratory investigation of the donor and recipients are strongly recommended. No active COVID-19 cases should be considered for transplant surgery. Deceased donor transplantation (even from recovered COVID-19 cases) needs careful consideration. 16 39

#### Residency Training

Urology residency training is compromised as the services are reduced and limited to nondeferrable cases. It has caused stress among medical students as all the medical examinations and academic rounds are currently postponed. 15 However, online presentation, discussion and telecast; simulation training; and virtual congressional meeting are actively fulfilling the gap and are popularly in the practice. 40 41 This pandemic is going to be there for quite a long time before any therapeutic or chemopreventive measure is discovered. Nassar and colleagues recommended the “functional-restructuring” of the residency program in the era of COVID pandemic to optimize the patient care and maintain the morale of residents. 42 Continued medical education to update the COVID-19 knowledge and skills (including critical care, palliative care, resource conservation, and ongoing clinical trials) is necessary. Residents need to care for personal safety and mental health; adopt telehealth and virtual learning; and supplement training with medical ethics, health policy, and global health. 43 Fifty-five percent of the urologist in training in France reported mental stress due to COVID pandemic. 44 Maintaining the healthy reserve of residents is necessary to mitigate burn out. 43

## Discussion

Guidelines Office Rapid Response Group (GORRG) of European Association of Urology (EAU) has divided the elective urological surgeries based on clinical harm down the timeline (low risk, no harm by 6 months; intermediate risk, no harm by 3 to 4 months, and high risk, no harm by 6 weeks). 45 The epidemiology of COVID-19 is rapidly changing and unpredictable. The effect of rescheduling the procedures can have a unique clinical outcome in each patient and is not easy to predict exactly for a span of timeframe. Risk of contracting and spreading the infection and benefiting the individual patient should be cautiously balanced. Prioritizing the urological care and selecting the modality of surgical intervention during this pandemic should be based upon available scientific evidence and local socioeconomic context. 10 16 31 We should not forget that the best available armamentarium for primary prevention is social distancing and personal protection equipment. The “test-trace-isolate” is another effective secondary prevention modality. Infectivity and mortality of SARS-CoV-2 virus need to be understood well. The 30-day postoperative outcomes were reported by the COVIDsurg collaborative from the international cohort (24 countries). They found 71.5% (806 out of 1,128) SARS-CoV-2 infection and 51.2% of pulmonary complications in the postoperative period with an overall mortality rate of 23.8%. The risk factors for complications were older age (>70 years), male gender, emergency surgeries, and malignant surgeries. 46 The overall worldwide mortality rate ranges from 2.3 to 5.6% and significantly higher in the age group >80 years (8%). 14 The SARS-CoV-2 virus was also isolated from stool and urine (6.9%) of the COVID positive patient. 47 48

Urological malignancy and the transplant candidates are most vulnerable to the disease progression as well as to the COVID-19 exposure. 16 39 The major triaging criteria for malignancy are its prognosis and provision of alternative treatment modalities and utilization of resources. 16 It requires informed clinical decisions and contingency. Active screening of the malignancy is not recommended. 22 The shortage of intravesical therapeutic agents for NMIBC (BCG - Bacillus Calmette–Guérin) can be addressed by using the alternative agent (mitomycin c) in selected cases or by reducing the dosage of the intravesical BCG to half to one-third. 49 The emergency conditions need to be managed efficiently and effectively without adding the extra risk of exposure and extra burden to health care resources. Human is both victim and vector of SARS-CoV-2. No age is immune. Pediatric urology too needs pandemic standard care. 24 50 Higher risk population group should avoid visiting the outpatient department. Malignant diseases are at higher risk than benign ( *p* = 0.001). 40 Telemedicine (video visit, virtual check-in, eVisit, and eConsult) not only provides zero risk urology services during a pandemic but also has the potential to grow into telemonitoring (cystoscopy, video-urodynamic, radiodiagnosis) and telesurgery (robotic surgery). 51 Based upon the risk grading, a study in Germany found that 54.1% of the patients in Germany were eligible (based on risk grading) and willing for tele-consultation for urology care that will ensure “contact-free continuity of care”. 25 Besides convenience, efficiency, and cost-utility the long-term quality health care (efficacy, safety, and equity) provided by the telehealth is not known. 51 All the urologists including the residents in urology should quickly learn and adopt the changing context of the health care system to serve patients and to save themselves. 42 43 44

We have included the current scientific evidence and academic articles in this review to make it comprehensive and simple. Evidence-based health care is the integration of research studies, clinical experiences, expert opinions, and patient values. The lower level (expert's opinion and consensus statements) of current evidence justifies the crisis standard approach. This is not the first and probably not the last pandemic of this world. Our review would provide important guidance and motivation for further scientific research to frame robust urology preparedness and response plan. Current evidence and guidelines are based on high-impact countries, mainly Europe and the United States (60%). This reserves room for reporting and publication bias and wider external validity. Further experiences and evidence from all corners of the world could help to draw more suitable and adoptable consensus among urologists worldwide. Observing and deferring what we currently assume nonessential could add new insight to the natural history of the disease.

## Conclusion

Balancing the crisis standard public health protection and individual clinical care is a big challenge. Triaging urology care is the best approach during a public health crisis. Informed decision making in health care has paramount importance during a pandemic. The COVID-19 pandemic has provided ample opportunity to learn, unlearn, and relearn the science. It is also time to plan a resilient health care system appropriate for the public health crisis which could afflict humankind time and again.
